# Estrogen receptor blockade and radiation therapy cooperate to enhance the response of immunologically cold ER+ breast cancer to immunotherapy

**DOI:** 10.1186/s13058-023-01671-y

**Published:** 2023-06-13

**Authors:** Kathleen A. O’Leary, Amber M. Bates, Won Jong Jin, Brian M. Burkel, Raghava N. Sriramaneni, Sarah E. Emma, Erin J. Nystuen, Elizabeth G. Sumiec, Suzanne M. Ponik, Zachary S. Morris, Linda A. Schuler

**Affiliations:** 1 Department of Comparative Biosciences, School of Veterinary Medicine, University of Wisconsin, University of Wisconsin-Madison, Madison, WI USA; 2 Department of Human Oncology, School of Medicine and Public Health, University of Wisconsin, University of Wisconsin-Madison, Madison, WI USA; 3 Department of Cell and Regenerative Biology, School of Medicine and Public Health, University of Wisconsin, University of Wisconsin-Madison, Madison, WI USA; 4 University of Wisconsin Carbone Cancer Center, University of Wisconsin-Madison, Madison, WI USA

**Keywords:** ER+ breast cancer, Anti-estrogen, Radiation therapy, Immunotherapy, Fulvestrant

## Abstract

**Background:**

Most patients with estrogen receptor positive (ER+) breast cancer do not respond to immune checkpoint inhibition (ICI); the tumor microenvironment (TME) of these cancers is generally immunosuppressive and contains few tumor-infiltrating lymphocytes. Radiation therapy (RT) can increase tumor inflammation and infiltration by lymphocytes but does not improve responses to ICIs in these patients. This may result, in part, from additional effects of RT that suppress anti-tumor immunity, including increased tumor infiltration by myeloid-derived suppressor cells and regulatory T cells. We hypothesized that anti-estrogens, which are a standard of care for ER+ breast cancer, may ameliorate these detrimental effects of RT by reducing the recruitment/ activation of suppressive immune populations in the radiated TME, increasing anti-tumor immunity and responsiveness to ICIs.

**Methods:**

To interrogate the effect of the selective estrogen receptor downregulator, fulvestrant, on the irradiated TME in the absence of confounding growth inhibition by fulvestrant on tumor cells, we used the TC11 murine model of anti-estrogen resistant ER+ breast cancer. Tumors were orthotopically transplanted into immunocompetent syngeneic mice. Once tumors were established, we initiated treatment with fulvestrant or vehicle, followed by external beam RT one week later. We examined the number and activity of tumor infiltrating immune cells using flow cytometry, microscopy, transcript levels, and cytokine profiles. We tested whether fulvestrant improved tumor response and animal survival when added to the combination of RT and ICI.

**Results:**

Despite resistance of TC11 tumors to anti-estrogen therapy alone, fulvestrant slowed tumor regrowth following RT, and significantly altered multiple immune populations in the irradiated TME. Fulvestrant reduced the influx of Ly6C+Ly6G+ cells, increased markers of pro-inflammatory myeloid cells and activated T cells, and augmented the ratio of CD8+: FOXP3+ T cells. In contrast to the minimal effects of ICIs when co-treated with either fulvestrant or RT alone, combinatorial treatment with fulvestrant, RT and ICIs significantly reduced tumor growth and prolonged survival.

**Conclusions:**

A combination of RT and fulvestrant can overcome the immunosuppressive TME in a preclinical model of ER+ breast cancer, enhancing the anti-tumor response and increasing the response to ICIs, even when growth of tumor cells is no longer estrogen sensitive.

**Supplementary Information:**

The online version contains supplementary material available at 10.1186/s13058-023-01671-y.

## Background

Breast cancer is the second leading cause of cancer-related deaths for women, and cancers that express estrogen receptor alpha (ER+) comprise about 70% of all breast cancers [1, 2]. Estrogen is a major driver of growth for many of these cancers, and fortunately, surgery and adjuvant therapies directed at ER successfully treat many of these patients. However, multiple mechanisms lead to resistance to anti-estrogens, and as many as 20% of these patients develop therapy-resistant recurrences, accounting for the majority of breast cancer related deaths [2, 3]. Additional therapeutic approaches are needed for these patients.

Immunotherapies have demonstrated exciting potential for some malignancies. In patients with immunologically “hot” tumors, which are characterized by a pre-existing but exhausted adaptive anti-tumor immune response, inhibitors of immune checkpoints such as programmed death-1 (PD-1), can block inhibitory interactions to overcome T cell exhaustion, thereby restoring anti-tumor activity and introducing the potential for durable tumor regression [4]. However, clinical benefits from these approaches in patients with ER+ breast cancers have been limited. In general, these tumors have low rates of somatic mutation, contain few infiltrating lymphocytes, and express low levels of PD-L1, indicators of immunologically “cold” cancers [5, 6]. Within the context of breast cancers, ER+ cancers exhibit less abundant immune infiltrates and fewer somatic mutations with concomitant reduced potential for tumor neo-antigens, compared to HER2+ and triple negative breast cancers (TNBC) [7, 8]. Consistently, patients with ER+ tumors respond more poorly to inhibition of the PD-1 axis than other breast cancer subtypes [9, 10].

Local radiation therapy (RT) is one way to combat the low immunogenicity of “cold” cancers.

RT initiates dynamic changes that can augment the anti-tumor immune response by multiple mechanisms, including: (1) induction of immunogenic tumor cell death and release of tumor-specific antigens, (2) increased expression of immune susceptibility markers on surviving tumor cells (e.g., cGAS-STING activation of type I IFN response), (3) local release of inflammatory cytokines and damage-associated molecular patterns which increase immune cell trafficking and activation, and 4) temporary local depletion of RT-sensitive immune lineages including suppressor and effector lymphocytes [11–13]. Together, these actions have been observed to enhance the response to immune checkpoint inhibitors (ICIs) [14–16]. Yet the complex actions of RT in the tumor environment also include those that impede anti-tumor immunity, including recruitment and activation of myeloid derived suppressor cells (MDSCs) and immunosuppressive regulatory T cells (Tregs) [11–13, 17]. In a Phase II clinical trial, RT alone was not sufficient to sensitize advanced ER+ breast cancers to the ICI, α-PD-1 [18].

It is well recognized that estrogen has complex effects on inflammation. Multiple immune cell subpopulations express estrogen receptors, albeit at lower levels than classic reproductive targets, and both myeloid and lymphoid lineages are estrogen responsive [7, 19–22]. Anti-estrogens have been shown to reduce tumor MDSCs and Tregs in murine models of ER negative ovarian and breast cancers [23, 24]. Recently, anti-estrogens have been shown to shift macrophage polarization toward a M1 inflammatory phenotype in preclinical models of ER negative melanoma [25]. Together, these observations suggest that anti-estrogen treatment, the standard of care for patients with ER+ breast cancer, may complement RT by reducing recruitment/ activity of suppressive immune cell populations, resulting in increased anti-tumor immunity and responsiveness to ICIs.

Here we interrogated the effect of the selective estrogen receptor downregulator (SERD), fulvestrant, in conjunction with RT in a relevant immunocompetent murine model of anti-estrogen resistant ER+ breast cancer [26]. Our studies demonstrate that fulvestrant slows regrowth of mammary TC11 tumors following RT, and significantly immunomodulates the dynamic irradiated tumor microenvironment (TME). Fulvestrant altered the numbers and/or activity of multiple immune subpopulations. It significantly reduced the RT-induced influx of cells with surface markers of MDSCs (Ly6C+Ly6G+) [27], increased indicators of macrophage inflammatory activity, and reduced FOXP3+Tregs to augment the ratio of CD8+ to FOXP3+ T cells, culminating in rising indicators of T cell activation. Furthermore, in contrast to the lack of effect of ICIs in animals treated with RT alone, co-treatment with fulvestrant and RT reduced tumor size and prolonged survival in concert with the individual ICIs, anti-PD-L1 and anti-CTLA-4. Our findings provide mechanistic insight into the complementary actions of these therapeutic approaches and point to new opportunities for further preclinical and clinical investigation.

## Methods

### Reagents

Fulvestrant was provided by AstraZeneca. Anti-murine PD-L1 antibody (α-PDL1; clone 10F.9G2) was purchased from BioXcell (Lebanon, NH). Anti-murine CTLA-4 antibody (α-CTLA-4, clone 9D9, IgG2c) was produced by NeoClone (Madison, WI). Antibodies utilized for immunohistochemistry and flow cytometry are listed in Additional file 1: Tables S1 and S2.

### Murine model and treatments

All animal studies were approved by the Institutional Animal Care and Use Committee at the University of Wisconsin–Madison. The murine ER+ mammary cancer cell line, TC11, was derived from a spontaneous prolactin-induced tumor that developed in the NRL-PRL model in the FVB/N genetic background [26, 28, 29]. Tumors in this model do not depend on estrogen for growth and are resistant to anti-estrogens, similar to many advanced clinical ER+ breast cancers, but none-the-less respond to estrogen activity with changes in gene expression (Additional file 2: Fig. S1A) [26, 28]. These features enable examination of anti-estrogen actions independent of effects on tumor growth. 15,000 TC11 cells were orthotopically transplanted into the caudal mammary fat pads of syngeneic intact female mice aged 8 to 10 weeks old. Mice were randomized into treatment groups, and three weeks after transplantation, treatments were initiated. Mice were monitored daily, and tumor sizes were measured twice weekly with calipers by blinded investigators (tumor volumes calculated as the largest diameter × (the smallest diameter)^2^ × 0.4). When recipient mice reached end stage (mammary tumors 1500 mm^3^ in volume or evidence of discomfort and veterinary recommendation), animals were humanely euthanized and tissues collected.

The SERD, fulvestrant, inactivates and degrades the ER, and is approved for patients with advanced ER+ breast cancer whose disease has progressed on first line anti-estrogens [30]. As shown in the schematic experimental designs for each figure, treatments with fulvestrant, (250 mg/kg sc weekly) or peanut oil vehicle (control) were initiated three weeks after transplantation, when tumors were palpable. Physiologic efficacy of fulvestrant was confirmed by measurement of uterine weights (Additional file 2: Fig. S1B). For some experiments, a single dose of external beam radiation (RT, 8 Gy) was delivered to the tumor on day 28 (one week following the initial fulvestrant dose when tumors were about 150 mm^3^) or 3 doses on days 27, 28, 29 post transplantation (8 Gy × 3), using an Xrad320 (Pxi) irradiator (Precision X-Ray, Inc., North Branford, CT, USA). Normal tissues were shielded during RT using custom-made lead blocks. Some cohorts were injected intraperitoneally with α-PDL1 or α-CTLA-4 on days 31, 34 and 37 after transplantation (100 μL, 1 mg/mL).

### Immunohistochemical staining

A portion of mammary tumors were either fixed in 10% neutral buffered formalin and embedded in paraffin (FFPE) or embedded in optimal cutting temperature compound. Chromogenic immunohistochemistry was performed as previously described [29], using the antibodies listed in Additional file 1: Table S1A. For some experiments, CD8a+ or Ly6C+/Ly6G+ cells were quantified by counting 100 cells from 5 images/tumor from 5 mice/group.

Multispectral immunofluorescence was performed on FFPE sections using the Manual Opal 7-color kit (Akoya Biosciences NEL811001KT), and the antibodies listed in Additional file 1: Table S1B. Prior to multiplexing, each antibody was validated using conventional immunohistochemistry and monoplex immunofluorescence staining. The specimens were then counterstained with a spectral DAPI. The multiplexed slides were imaged using a Leica Thunder imaging system with a Dmi8 microscope base, an 8-line LED, and a DFC9000 GT scMOS camera. Slides were initially scanned at 20X for a tiled overview of the tissue, and then stacks of ten regions of interest were collected at Nyquist in each condition. Illumination levels were set to maximize dynamic range of each channel, and were held constant across conditions. Stacks were deconvolved with the Small Volume Computational Clearing module in the LAS X software. A single plane from the center of the Z-stack was selected for display purposes. LUT thresholds were set based on cellular morphological features and high expression. For each channel, display thresholds were held constant across conditions.

### Flow cytometry analysis

Portions of some tumors were processed for flow cytometry as previously described [31]. Single cell suspensions were stained with surface antibodies (Additional file 1: Table S2) and then fixed using the eBioscience FOXP3 fixation/permeabilization kit. UltraComp Beads eBeads (Invitrogen) were used for compensation and fluorescence minus one (FMO) methodology to determine gating. Flow cytometry was performed on an Attune Nxt flow cytometer (ThermoFisher), and compensation matrix and data were analyzed using FlowJo software. Tumor infiltrating innate and adaptive immune cell subpopulations were quantitated using the gating strategy shown in Additional file 2: Fig. S2A and B, respectively.

### Multiplex cytokine immunoassay

To determine effects of RT and fulvestrant on the cytokines in the TME, tumors were harvested from mice 53 days after transplantation (24 days after the final RT dose), and lysates were analyzed by a multiplex immunoassay of 30 cytokines and chemokines (MILLIPLEX MAP Mouse Cytokine/Chemokine Magnetic Bead Panel, Millipore) as described [31]. Data were log and Z-transformed, and a heatmap was created using the NGM heat map builder.

### Gene expression analysis

Total RNA was isolated, cDNA was synthesized, and quantitative real-time polymerase reactions were carried out using PowerUp SYBR Green qPCR Master Mix (Life Technologies) and the QuantStudio 6 Pro Real-Time PCR System (Applied Biosystems) as described [31]. Primer information can be found in Additional file 1: Table S3A. For other experiments, qPCR was performed using Taqman Fast Advanced Master Mix and predesigned Taqman Gene Expression Assays (Additional file 1: Table S3B). Fold changes were calculated using the ∆∆Ct method relative to the expression in the oil (in vivo) or ethanol (in vitro) controls, using *Hprt* as an endogenous control.

### In vitro studies

TC11 cells were cultured in RPMI-1640 with 10% heat-inactivated fetal bovine serum and 1% penicillin–streptomycin at 37° C in a humidified incubator with 5% CO_2_. To examine the time course of responses, cells were plated at varying concentrations. After 24 h to allow adherence, 8 Gy of external beam radiation was delivered to the cells using a RS225 Cell Irradiator (Xstrahl) (day 0). The media was changed immediately, and ethanol vehicle or 100 nM fulvestrant was added. Media containing treatments were changed every 3 days, and cells were harvested after 1, 3, and 5 days for isolation of total RNA and subsequent qPCR analyses as described above.

In vitro clonogenic assays were performed to examine the effect of fulvestrant on radiosensitivity. TC11 cells were seeded at 5 × 10^5^ cells/60 mm tissue culture plate. After allowing the cells to adhere overnight, media containing 1 µM of fulvestrant or an equal volume of ethanol was added. After 24 h, the cells were irradiated with 0 Gy (sham RT), 2 Gy, 4 Gy, or 8 Gy using a RS225 Cell Irradiator (Xstrahl). After irradiation, cells were harvested and replated for analysis of clonogenic survival. Surviving colonies were stained with crystal violet, and numbers of colonies containing > 50 cells were counted to determine plating efficiency and the fractions of surviving cells.

### Statistical analysis

Data are displayed as means ± standard error of mean (SEM) unless otherwise stated. Sample sizes were based on previous experience. For survival analysis, Kaplan–Meier curves were generated, and the Mantel Cox log-rank test was used to detect the presence of differences within an experiment. If significant differences were observed, log-rank pairwise comparison tests were conducted to compare overall survival between treatment groups. One-way ANOVA followed by post-hoc multiple comparisons tests with Tukey adjustment for p-values were used to determine the statistical significance among treatment groups. Tumor sizes among treatment groups were compared using unpaired t tests or ANOVA, as described in the figure legends. All analyses were performed in GraphPad Prism (v.8.4.3) or R (v.4.0.2). P-values less than 0.05 were considered significant.

## Results

**Fig. 1:**
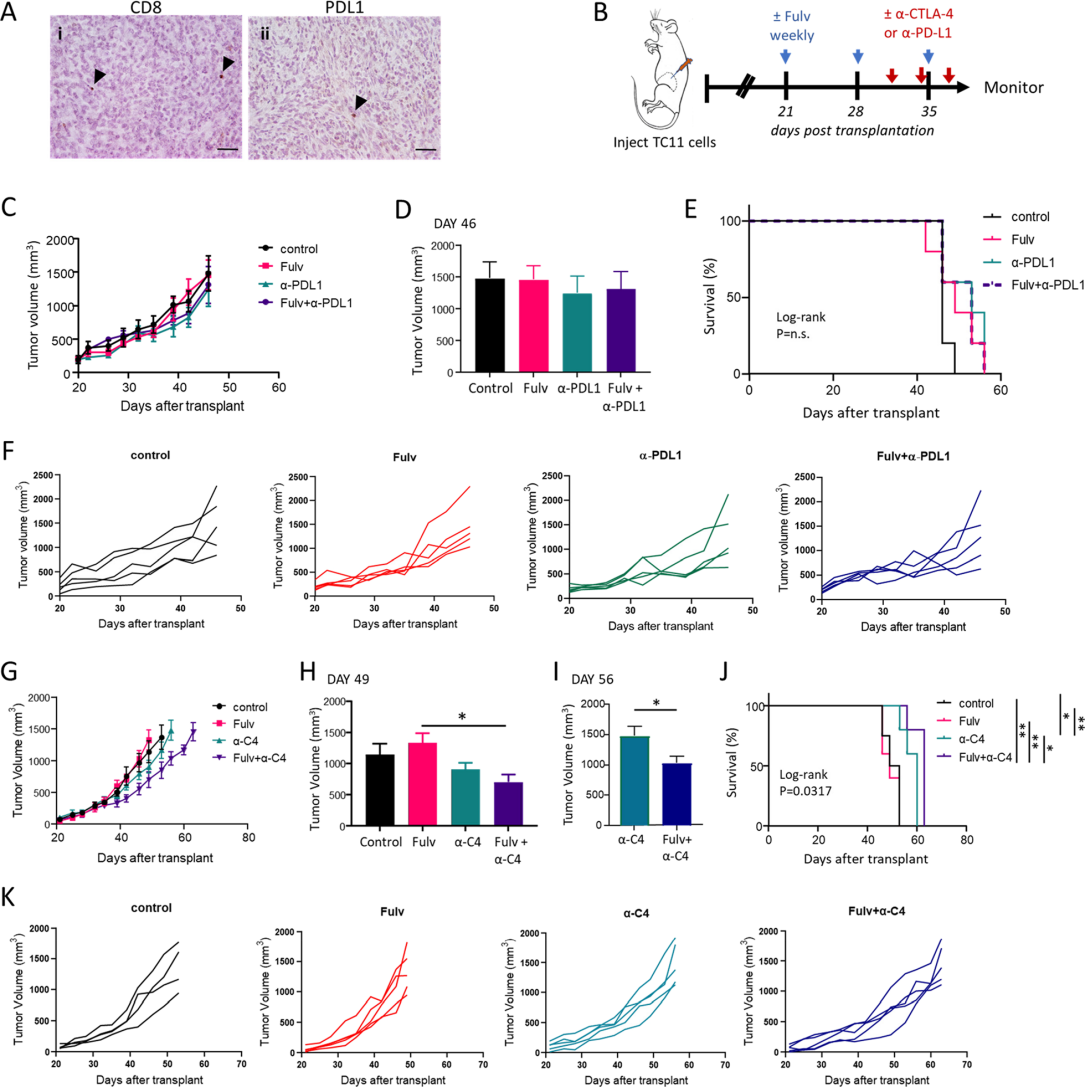
TC11 ER+ tumors are immunosuppressed, anti-estrogen resistant and poorly responsive to immune checkpoint inhibitors. **A** TC11 cells were orthotopically transplanted to the caudal mammary fat pads of syngeneic FVB/N young adult females. i. immunostaining for CD8 (arrowheads); ii. immunostaining for PD-L1 (arrowheads). Original magnifications ×200; scale bars = 50 µm. **B** Schematic of treatment timeline. Three weeks after transplantation, when tumors were palpable, weekly treatments with fulvestrant (Fulv) or oil vehicle control were initiated. After ten additional days, three doses of ICIs (α-PDL1, α-CTLA-4) were administered (days 31, 34 and 37 after transplantation). Tumor sizes were measured biweekly. **C–F** Neither fulvestrant nor α-PDL1, alone or in combination, impacts tumor growth (**C, D, F**) or survival (**E**). **C** Tumor volumes (mean ± SEM, n = 6 for single treatments, n = 9 for Fulv+α-PDL1). **D** Comparison of mammary tumor volumes on day 46 after transplantation, when all animals in all treatment groups were alive. One way ANOVA identified no significant differences. **E** Kaplan Meier analysis of survival (log-rank test, n.s., no significant differences). **F** Tumor growth curves in individual mice shown in (**C**). **G-K** α-CTLA4 (α-C4) modestly reduces tumor growth and increases survival, which is significantly improved in combination with fulvestrant. **G** Tumor volumes (mean ± SEM, n = 4–5). **H** Comparison of mammary tumor volumes on day 49 after transplantation, when no animals had reached end stage. Differences among treatments were determined by one way ANOVA, followed by Tukey post tests (*, *p* < 0.05). **I** Comparison of mammary tumor volumes on day 56 after transplantation, when only animals treated with α-CTLA4 and those treated with Fulv/α-CTLA4 combination were alive. Unpaired t test (*, *p* < 0.05). **J** Kaplan Meier analysis of survival (log rank tests, *, *p* < 0.05; **, *p* < 0.01). **K** Tumor growth curves in individual mice shown in (**G**)

Among breast cancer subtypes, ER+ tumors contain lower numbers of tumor infiltrating lymphocytes and are less responsive to ICIs than TNBCs [5–8]. Given that estrogen exerts complex effects on inflammation and immune cell differentiation and has been shown to influence intratumoral immune cell populations [7, 19–25], we hypothesized that SERDs, such as fulvestrant, could modulate the tumor immune microenvironment of these cancers. Transplanted ER+ TC11 cells provide an immunocompetent model to investigate the interactions of estrogen inhibition, ICIs, and RT in aggressive ER+ breast cancer (Additional file 2: Fig. S1Ai, ii) [26]. As shown in Fig. 1A, ER+ TC11 tumors that result from orthotopic transplantation contain few intratumoral CD8+ cells, and express little PD-L1, features of immunologically "cold" tumors. To examine the effect of anti-estrogens, we initiated treatments with the SERD, fulvestrant, beginning 3 weeks after transplantation (Fig. 1B). Although fulvestrant reduced uterine weight (Additional file 2: Fig. S1B), it did not inhibit growth of these anti-estrogen resistant mammary cancers [26], nor did it increase survival (Fig. 1C-K), similar to resistant advanced clinical ER+ cancers. To determine any interaction of anti-estrogens with ICIs, we administered 3 doses of either α-PDL1 or α-CTLA-4 (α-C4). Similar to advanced clinical breast cancers [9, 10], inhibition of the PD axis as a monotherapy had little effect on tumor growth or survival (Fig. 1C-F), consistent with the low levels of infiltrating CD8+ T cells and PD-L1 expression in these immunosuppressed tumors. Intriguingly, fulvestrant in combination with the ICI, α-CTLA-4, modestly but significantly reduced tumor growth (Fig. 1G–I, K). In addition, α-CTLA-4 alone significantly increased survival, which was significantly prolonged by co-treatment with fulvestrant (Fig. 1J).

**Fig. 2:**
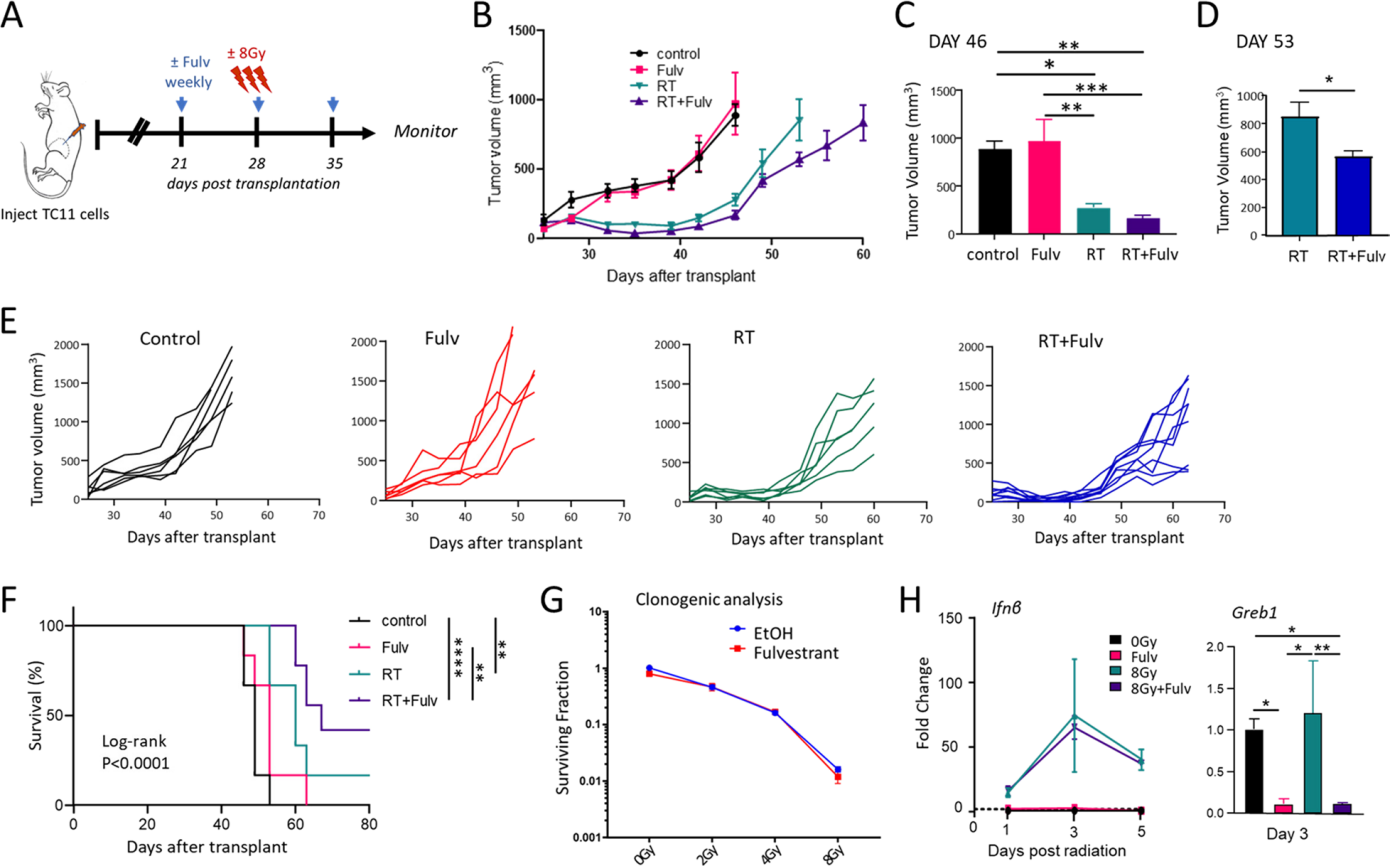
Anti-estrogen and radiation in combination slows the growth of TC11 ER+ mammary tumors. **A** Schematic of treatment timeline. FVB/N mice with TC11 tumors in the caudal mammary fat pads received weekly treatments with fulvestrant (Fulv) or oil control, initiated 3 weeks after transplantation as in Fig. 1, and/or 3 doses of RT (8 Gy administered on days 6, 7, and 8 following initiation of Fulv or oil treatments). **B** Tumor size was measured biweekly (mean ± SEM, n = 6 for single treatments, n = 9 for RT+Fulv). **C** Comparison of mammary tumor volumes in animals receiving RT and RT+Fulv on day 46 after transplantation, the last time when animals in all treatment groups were still alive. Differences among treatments were determined by one way ANOVA, followed by Tukey post tests, *, *p* < 0.05; **, *p* < 0.01; ***, *p* < 0.001. **D** Comparison of mammary tumor volumes in animals receiving RT and RT+Fulv on day 53 after transplantation, the last time when the RT-treated group was alive. Unpaired t test, *, *p* < 0.05. **E** Tumor growth curves in individual mice shown in (**B**). **F** Kaplan Meier analysis of survival (log-rank tests, **, *p* < 0.01; ****; *p* < 0.0001). **G** Fulvestrant does not affect the clonogenic response to RT in TC11 cells in vitro. 24 h after plating, TC11 cells were treated ± Fulv for 24 h, followed by a single dose of increasing RT (0–8 Gy). Clonogenic analyses were performed as described in the Materials and Methods. **H** Fulvestrant does not alter the type I IFN response following RT in vitro, which has been reported to occur downstream of cGAS/STING activation, but this treatment strongly reduces transcripts for *Greb1*, a well characterized estrogen target gene. TC11 cells were treated ± 8 Gy RT, ± Fulv for the times shown, and transcripts analyzed by qPCR as described in the Materials and Methods. Differences among treatments were determined by one way ANOVA, followed by Tukey post tests (*, *p* < 0.05; **, *p* < 0.01)

RT induces dynamic changes in the TME which impact anti-tumor immunity, and a growing literature demonstrates that RT can cooperate with ICIs to reduce tumor growth in many preclinical models and possibly in some clinical settings [11, 14–16]. However, RT not only increases anti-tumor immunity, but also increases immunosuppressive immune cell subpopulations such as MDSCs and Tregs [11–13, 17]. The ability of anti-estrogens to reduce expansion and activation of these immune populations [23, 24], and the positive interaction of fulvestrant with α-CTLA-4 in this ER+ model (Fig. 1G-K) led us to hypothesize that anti-estrogens may favorably immunomodulate the irradiated ER+ TME. To examine this possibility, we administered RT to established TC11 tumors, beginning one week after initiation of fulvestrant treatment (Fig. 2A). As shown in Fig. 2B, C, fulvestrant modestly prolonged the regrowth delay induced by RT; at day 53 after transplantation, tumors co-treated with RT and fulvestrant were significantly smaller than those treated with RT alone (Fig. 2D-E), resulting in a trend towards improved survival with this treatment combination (*p* = 0.062, Fig. 2F).

In order to evaluate if the interaction between RT and fulvestrant in vivo is in part the result of action on tumor cells alone, we examined responses of TC11 cells in vitro. We performed clonogenic assays to examine the effects of fulvestrant on the intrinsic radiosensitivity of these tumor cells. As shown in Fig. 2G, fulvestrant did not alter the surviving fraction of TC11 cells, in contrast to the anti-estrogen responsive human breast cancer cell lines, MCF7 and T47D [32]. The anti-tumor effects of RT have been shown to be at least partially dependent on the immune response in vivo, and activation of a type I interferon (IFN) response by the cGAS/STING pathway has been implicated as a critical step in mediating this anti-tumor immune response [33–36]. We therefore examined the effect of fulvestrant on the RT-induced STING pathway. As shown in Fig. 2H, RT induced a rapid increase in transcripts for the type I IFN, IFNβ. Fulvestrant did not alter this response under these conditions in vitro, despite efficient inhibition of transcription of the ER target gene, *Greb1*. Together, these data suggest that the observed interaction between RT and fulvestrant to reduce tumor growth in vivo may not be tumor cell autonomous.

**Fig. 3:**
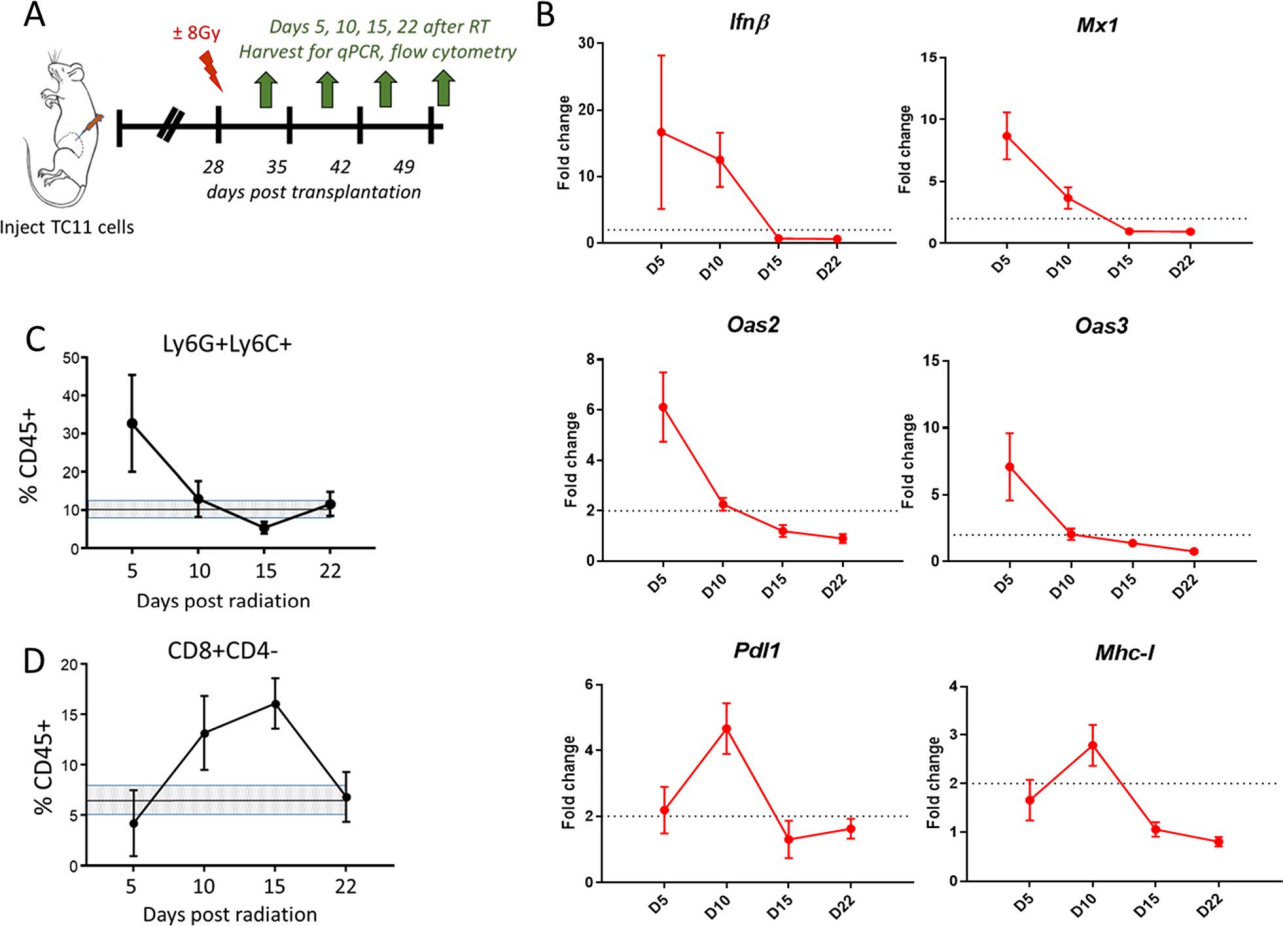
In TC11 tumors, RT induces dynamic changes in markers for a type I IFN response and immune susceptibility, and immune cell subpopulations. **A** Schematic of treatment timeline. TC11 tumors in the caudal mammary fat pads were treated ± a single dose of RT (8 Gy) 28 days (4 weeks) after tumor cell transplantation (tumors about 150mm^3^). Cohorts of mice were euthanized 5, 10, 15, and 22 additional days after RT, and the tumors were harvested for qPCR and flow cytometry analyses. **B** Relative levels of transcripts for genes associated with a type I interferon response (*Ifnβ, Mx1, Oas2, Oas3*) and immune susceptibility (*Pdl1, Mhc-1*) are shown. (Mean ± SEM, n = 5). A twofold increase (dotted line) or greater is considered significant. **C, D** Relative proportions of intratumoral CD45+ immune cells that expressed Ly6G+Ly6C+(**C**) or CD8+CD4− (**D**), analyzed by flow cytometry. (Mean ± SEM, n = 5). The mean levels ± SEM in tumors which were not irradiated are shown by the shaded lines

The time course of immune responses initiated by RT in vivo can vary among tumor types [37]. We therefore examined the kinetics of responses to RT delivered to TC11 tumors (Fig. 3A). As shown in Fig. 3B, RT induced a rapid type I IFN response, and activation of downstream IFN response genes (*Mx1, Oas2, Oas3*) by day 5 after RT, which then fell to non-radiated control levels by day 15. Transcripts indicating modified immune susceptibility (*Pdl1, Mhc1*) peaked at day10 post-RT, after which they also declined (Fig. 3B). RT also induced changes in immune cell subpopulations. By day 5, Ly6C+Ly6G+ immune cells, which have been reported to include MDSCs [27], were elevated in the TME, and declined thereafter (Fig. 3C), similar to other models [17]. In contrast, a RT-induced increase in CD8+ T cells was not evident until day 10 (Fig. 3D). We therefore further examined the interaction of fulvestrant with RT on days 5 and 10 post RT, when infiltration of these myeloid and adaptive T cell subpopulations was respectively maximal.

**Fig. 4:**
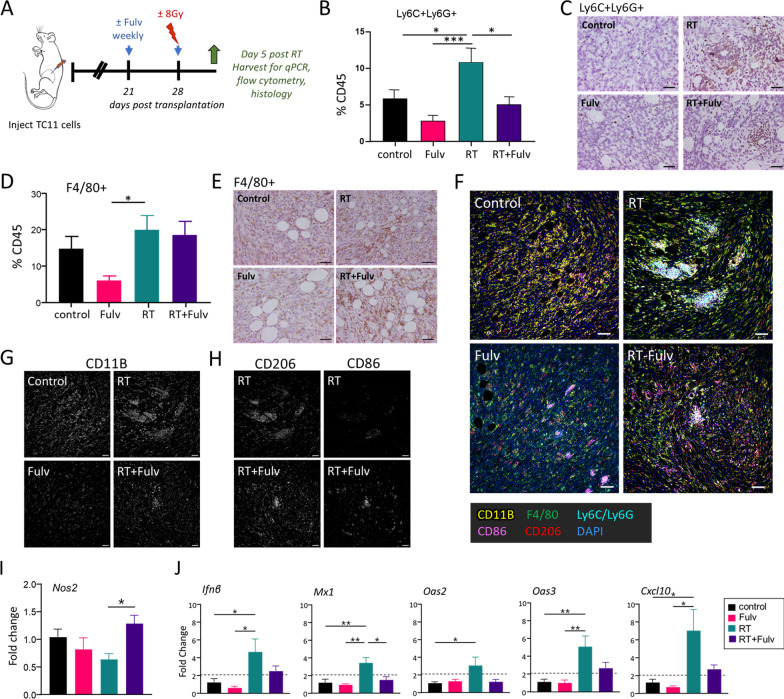
Fulvestrant modulates RT-induced changes in myeloid immune cell populations. **A** Schematic of treatment timeline. TC11 cells were transplanted to the caudal mammary fat pad as in Fig. 1. Fulvestrant (Fulv) / oil control treatments were initiated 3 weeks after transplantation, and/or a single dose of RT (8 Gy) was administered 1 week following the first Fulv/ vehicle treatment. TC11 tumors were collected at day 5 after RT for flow cytometry, immunohistochemistry, and qPCR analyses. **B** Relative proportions of tumor CD45+ immune cells that expressed Ly6C/Ly6G at day 5 after RT, analyzed by flow cytometry (mean ± SEM, n = 9). Differences among treatments were determined by one way ANOVA, followed by Tukey post tests. *, *p* < 0.05; ***, *p* < 0.001. **C** Tumor infiltrating Ly6C+/Ly6G+cells visualized by immunohistochemical staining, representative sections. Original magnifications, × 200; scale bars = 50 µm. See Additional file 2 Fig. S3 for quantitation. **D** Relative proportions of tumor CD45+ immune cells that expressed F4/80 at day 5 after RT, analyzed by flow cytometry (mean ± SEM, n = 9–10). Differences among treatments were determined by one way ANOVA, followed by Tukey post tests. *, *p* < 0.05. **E** Tumor infiltrating F4/80+ cells shown by immunohistochemical staining, representative sections. Original magnifications, × 200; scale bars = 50 µm. **F** Multispectral immunofluorescence staining of myeloid cells in the TME, labeled as shown. Original magnifications, × 200; scale bars = 50 µm. **G** CD11B signal in sections shown in (**G**). Original magnifications, × 200; scale bars = 50 µm. **H** CD206 and CD86 signals in tumors from animals treated with RT and RT+Fulv in sections shown in (**G**). Original magnifications, × 200; scale bars = 50 µm. **I** Relative levels of *Nos2* transcripts, an indicator of M1 activity. Mean ± SEM; n = 5. Differences among treatments were determined by one way ANOVA, followed by Tukey post tests. *, *p* < 0.05. **J** Relative levels of transcripts for genes associated with a type I interferon response (mean ± SEM, n = 4–5). Fulvestrant reduced activation of a type I IFN response by RT in vivo, in contrast to treatment of isolated TC11 cells in vitro (Fig. 2H), suggesting an effect on either the contents of the TME or on the response of the TME to RT. Differences among treatments were determined by one way ANOVA, followed by Tukey post tests. *, *p* < 0.05; **, *p* < 0.01

We treated the animals as shown in Fig. 4A, and examined tumors 5 days after RT, the peak time of the RT-induced myeloid cell influx and type I IFN response (Fig. 3B, C). At this time, co-treatment with fulvestrant and RT significantly reduced the RT-induced increase in Ly6C+Ly6G+ cells, evident by flow cytometry and immunohistochemistry (Fig. 4B, C, Additional file 2: Fig. S3). As shown in Fig. 4C, a few isolated Ly6C+Ly6G+ cells were scattered in the tumor parenchyma in control mice and those treated with fulvestrant as a single treatment. In response to RT, more Ly6C+Ly6G+ immune cells were evident, many in small clusters throughout the tumor. Although co-treatment with fulvestrant reduced the numbers of these cells relative to RT alone (Fig. 4B, Additional file 2: Fig. S3), it did not alter this spatial pattern (Fig. 4C). Macrophages are abundant in TC11 tumors (Fig. 4D), similar to clinical breast cancers [38–40]. TC11 tumors in mice treated with fulvestrant as a monotherapy contained fewer F4/80+ cells than those treated with RT alone, but fulvestrant in combination with RT did not alter the number of F4/80+ cells in tumors compared to RT as a monotherapy (Fig. 4D, E).

In order to further investigate the effects of RT and fulvestrant on myeloid immune cell subpopulations, we used multiplex immunofluorescence to visualize all myeloid cells (CD11B+), MDSCs (Ly6C+/Ly6G+), macrophages (F4/80+) and cells expressing markers of macrophage polarization (CD86, commonly associated with macrophages demonstrating M1-like polarization; CD206, commonly associated with M2 macrophages). These studies revealed the rich myeloid content of these tumors and the heterogeneous spatial distribution of these cells (Fig. 4F). CD11B+ myeloid cells were abundant throughout the tumor mass (Fig. 4F, G), similar to clinical tumors [39]. Consistent with the trend for fulvestrant to reduce intratumoral content of these immune subpopulations (Fig. 4B–E), tumors from animals treated with fulvestrant displayed lower CD11B staining than controls, and tumors from mice co-treated with RT+ Fulv exhibited lower CD11B staining than tumors treated with RT alone, except for islands of Ly6C+Ly6G+ cells (Fig. 4G). F4/80+ macrophages were plentiful, particularly in RT-treated tumors (Fig. 4F), consistent with the observations in Fig. 4D, E.

To further examine the effect of fulvestrant on myeloid activity in the radiated TME, we focused on expression of the macrophage polarization markers, CD86 and CD206, in tumors receiving RT alone and RT+ Fulv co-treatment. As shown in Fig. 4H, cells expressing CD206 were abundant in RT-treated tumors independent of fulvestrant treatment. However, co-treatment with RT+ Fulv increased CD86 expression, and some clusters of cells appeared to express both markers, suggesting that fulvestrant shifts the functional status of this plastic immune cell population in the irradiated TME, consistent with the reported ability of estrogen to modulate macrophage polarization [25]. In further support of a shift toward M1 activity, fulvestrant co-treatment with RT significantly elevated *Nos2* transcripts, a marker for M1 macrophages, in the bulk tumor, compared to tumors treated with RT alone (Fig. 4I)**.** Taken together, these data indicate that fulvestrant significantly modulates the effect of RT on the infiltration and activation of innate immune cell lineages within these ER+ mammary tumors.

Interestingly, in contrast to the inability of fulvestrant to directly antagonize the RT-induced type I IFN response in TC11 cells in vitro (Fig. 2H), addition of fulvestrant treatment to RT in vivo reduced intratumoral transcripts in the type I IFN pathway (Fig. 4J).

**Fig. 5:**
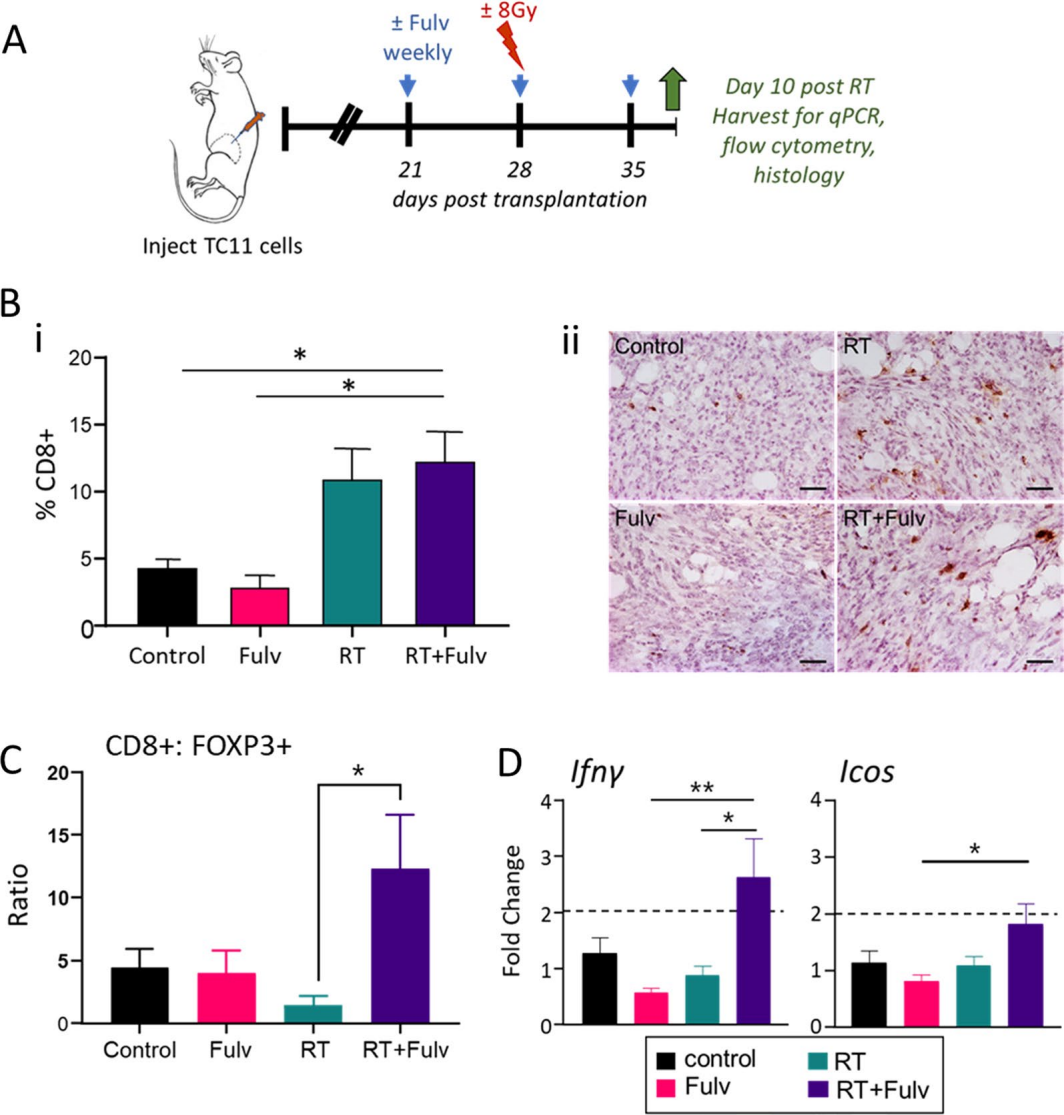
Co-treatment with fulvestrant increases the ratio of CD8+: FOXP3+ cells and markers of T cell activity in the TC11 TME. **A** Schematic of treatment timeline. TC11 cells were transplanted to the caudal mammary fat pad as in Fig. 1. Fulvestrant (Fulv)/ oil control treatments were initiated 3 weeks after transplantation, and/or a single dose of RT (8 Gy) was administered 1 week after initiation of fulvestrant treatments. TC11 tumors were collected at day 10 after RT for immunohistochemistry, flow cytometry and qPCR analyses. **B** Intratumoral CD8+ T cells at day 10 after RT were immunostained and quantified as described in the Materials and Methods. **i.** Quantification of intratumoral CD8+ infiltrate (mean ± SEM, n = 5). Differences among treatments were determined by one way ANOVA, followed by Tukey post tests. *, *p* < 0.05. **ii.** Intratumoral CD8+ cells shown by immunohistochemical staining, representative sections. Original magnifications, × 200; scale bar = 50 µm. **C** Ratio of CD8+: FOXP3+ cells in the tumor infiltrate. Proportions of CD8+ and FOXP3+ cells in CD45+ immune cells were determined by flow cytometry (mean ± SEM, n = 4). Differences among treatments were determined by one way ANOVA, followed by Tukey post tests. *, *p* < 0.05. **D** Relative levels of transcripts for genes reflecting T cell activity (mean ± SEM, n = 7–10). Differences among treatments were determined by one way ANOVA, followed by Tukey post tests. *, *p* < 0.05; **, *p* < 0.01

We examined the effects of fulvestrant on the adaptive immune response on day 10 following RT (Fig. 5A). As shown in Fig. 5B, RT significantly increased the density of CD8+ cells throughout the tumor mass, consistent with Fig. 3D, and co-treatment with fulvestrant did not alter this influx. Moreover, although the ratio of CD8+ to FOXP3+ cells in response to RT alone remained low, co-treatment with RT+Fulv reduced FOXP3+Tregs to significantly elevate this ratio (Fig. 5C). This was associated with significant increases in transcripts for IFNγ (Fig. 5D), consistent with increased T cell activation with the combination treatment. These results show that fulvestrant enhances some of the immunostimulatory effects of RT.

**Fig. 6:**
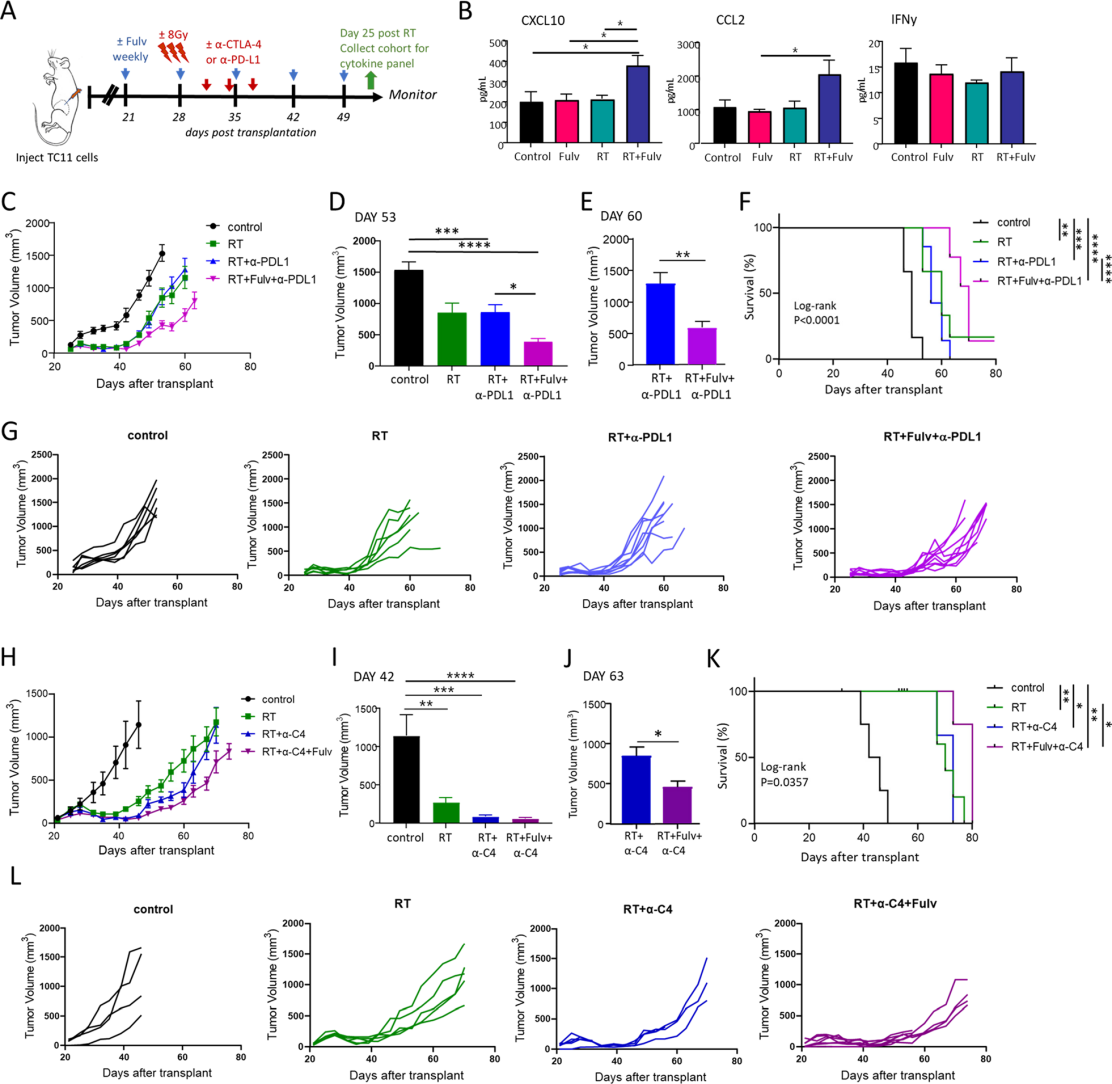
Fulvestrant in combination with RT improves the response to α-PDL1 and α-CTLA4, although tumor growth eventually resumes. **A** Schematic of treatment timeline. Three weeks after transplantation, treatment with fulvestrant (Fulv) or oil control was initiated. Beginning day 6 after initiation of Fulv/ vehicle treatments, tumors were administered ± 3 doses of RT (8 Gy) on three consecutive days (days 27, 28, and 29 after transplantation). After two additional days, three doses of ICIs (α-PDL1 or α-CTLA-4) were administered (days 31, 34 and 37 after transplantation). Tumors from one cohort of animals were collected on day 53 post transplantation (day 25 after RT, green arrow). Remaining mice were euthanized at end stage. **B** Levels of intratumoral cytokines 25 days after RT were determined by multiplex immunoassays (see also Additional file 2 Fig. S4). (Mean ± SEM, n = 5). Differences among treatments were determined by one way ANOVA, followed by Tukey post tests. *, *p* < 0.05. **C-F** Fulvestrant further slows growth of tumors and increases survival of mice co-treated with RT+α-PDL1. (N.B.: additional single and dual treatment response data are shown in Figs. 1 and 2). **C** Tumor volumes (mean ± SEM; n = 6 for single treatments, n = 9 for combination treatments). **D** Comparison of tumor sizes in mice receiving treatments in (**C**) at day 53 post transplantation, the latest time point when no animals had reached end stage. Differences among treatments were determined by one way ANOVA, followed by Tukey post tests. *, *p* < 0.05; ***, *p* < 0.001; ****, *p* < 0.0001. **E** Comparison of tumor sizes in mice receiving treatments in (**C**) at day 60 post transplantation, the latest time when animals receiving RT+α-PDL1 and RT+Fulv+α-PDL1 were alive. Differences between treatments were determined by unpaired t test. **, *p* < 0.01. **F** Kaplan Meier analysis of survival (log-rank tests, **, *p* < 0.01; ***, *p* < 0.001; ****; *p* < 0.0001). **G** Tumor growth curves in individual mice shown in (**C**). **H–L** Fulvestrant increases survival of mice co-treated with RT+α-CTLA-4 (α-C4). (N.B.: additional single and dual treatment response data are shown in Figs. 1 and 2). **H** Tumor volumes (mean ± SEM; n = 5 for all treatment groups except RT+α-C4+Fulv, where n = 7). **I** Comparison of tumor sizes receiving each treatment in (**H**) at day 42, the latest time point when no animals had reached end stage. Differences among treatments were determined by one way ANOVA, followed by Tukey post tests. **, *p* < 0.01; ***, *p* < 0.001; ****, *p* < 0.0001. **J** Comparison of tumor sizes receiving treatments in (**H**) at day 63 post transplantation, the latest time point when the majority of animals receiving RT+α-C4 and RT+Fulv+α-C4 were alive. (RT+α-C4, n = 3; RT+Fulv+α-C4, n = 4). Differences between treatments were determined by unpaired t test. *, *p* < 0.05. **K** Kaplan Meier analysis of survival (log-rank tests, *, *p* < 0.05; **; *p* < 0.01. RT+α-C4 vs. RT+α-C4+Fulv, *p* = 0.0502). **L** Tumor growth curves in individual mice shown in (**H**)

However, tumors in RT+Fulv co-treated animals eventually resumed growth (Fig. 2). To assess the cytokine environment in the TME during this period of regrowth, concentrations of intratumoral cytokines were examined by multiplex immunoassays at 25 days following RT (Fig. 6B; Additional file 2: Fig. S4). At this time, CXCL10 and CCL2 were significantly increased in the tumors of animals that received RT+Fulv (Fig. 6B); no other tested cytokines were consistently affected by the combination of these treatments (Additional file 2: Fig. S4). CXCL10 is produced by multiple cell types in the tumor environment, including monocytes and neutrophils, and promotes recruitment of T cells and natural killer cells; CCL2 is produced by monocytes and macrophages, and is an attractant for monocytes, tumor associated macrophages and natural killer cells [41]. These data show that anti-estrogen therapy in combination with RT can exert lasting immunomodulatory effects in the irradiated TME.

In contrast to the elevated *Ifnγ* transcripts in RT+Fulv co-treated animals on day 10 after RT (Fig. 5D), by day 25, IFNγ was no longer elevated in tumors of animals receiving the combination treatment (Fig. 6B), indicating immune exhaustion. We therefore asked whether fulvestrant could enhance or prolong the anti-tumor response to RT in combination with immune checkpoint inhibitors using the experimental design shown in Fig. 6A. In contrast to the minimal effects of ICIs when added to RT alone in this system, resembling clinical findings [18], fulvestrant added to RT+ α-PDL1 significantly delayed tumor regrowth (Fig. 6C–E, G), and prolonged survival (Fig. 6F). Similarly, addition of fulvestrant to RT+ α-CTLA4 (α-C4) also significantly slowed tumor growth and prolonged survival (Fig. 6H-L). Together, these findings show that fulvestrant can enhance anti-tumor immunity in combination with RT and ICIs in a hormone-therapy resistant ER+ breast cancer model.

## Discussion

ER+ breast cancers generally exhibit fewer neo-antigens and less lymphocytic infiltrate than HER2+ or TNBC subtypes, presenting a challenge that limits the potential for clinical benefit from immune checkpoint blocking therapies. RT has been shown to be a powerful tool to promote the influx of multiple immune cell populations and increase tumor neo-antigen cross presentation [11–13]. These processes can fuel anti-tumor immunity, although the dynamic and complex responses to RT also include recruited MDSCs and Tregs that can antagonize this effect [11–13, 17], and RT alone was not sufficient to sensitize advanced clinical ER+ breast cancers to immune checkpoint inhibition with α-PD-1 [18], as mimicked in our experimental model. Here we demonstrated that an anti-estrogen (fulvestrant), which is a standard of care treatment for ER+ breast cancer, can beneficially shift the immunologic landscape of the irradiated TME by significantly changing the numbers and activity of multiple immune cell populations, resulting in enhanced responses to ICIs, even when growth of these tumors does not respond to anti-estrogen therapy alone. In light of the mechanistic synergies between RT and fulvestrant to favorably immunomodulate the TME shown here and the clear clinical safety record of concurrent adjuvant and palliative RT with systemic anti-estrogens, our studies strongly support revisiting this combination along with ICIs in the large numbers of patients with advanced anti-estrogen resistant ER+ breast cancer. Given the size of this population, even modest therapeutic effects would be highly meaningful from a clinical perspective.

Consistent with the resistance of TC11 tumors to estrogen receptor antagonists [26] (and data herein), fulvestrant did not increase the intrinsic radiosensitivity of TC11 tumor cells in vitro, in contrast to other breast cancer cell lines that are dependent on estrogen for growth [32]. Fulvestrant also did not alter the RT-induced type I IFN response in TC11 cells in culture, despite potent antagonism of estrogen-driven transcription of a classic estrogen target gene. Rather, our data indicate that the favorable effects of fulvestrant in the context of RT were primarily tumor cell extrinsic. As a monotherapy, fulvestrant showed a trend to reduce several intratumoral immune populations, including Ly6C+/Ly6G+ and F4/80+ myeloid cells and FOXP3+ lymphocytes, compared to control recipients at varying stages of the estrous cycle. These effects are consistent with reports of altered estrogen activity in several clinical and experimental studies [23, 24, 42, 43]. However, fulvestrant-induced immunomodulatory effects were strikingly evident in the context of the dynamic changes in the TME initiated by RT. Fulvestrant significantly inhibited the RT-induced influx of immunosuppressive Ly6C+/Ly6G+ cells, illuminated by the mechanistic studies of estrogen-induced expansion, recruitment and activation of these cells [23]. Although fulvestrant did not reduce RT-induced infiltration of F4/80+ macrophages into irradiated tumors, it increased indicators of myeloid inflammatory activity, consistent with a relative rise in M1-like polarization. The direct inhibition of estrogen signaling in macrophages, recently reported to impact T cell activity in an experimental melanoma model [25], is likely to contribute to this shift. Fulvestrant did not alter the RT-induced influx of CD8+ T cells, but it reduced FOXP3+ cells, consistent with the reported ability of estrogen to promote Treg differentiation [22, 44], thus augmenting the ratio of CD8+ to FOXP3+ T cells. Direct actions on these ER+ immune cell populations would be influenced by the myriad of other estrogen responsive cells in the TME, which include not only the ER+ tumor cells, but also other non-immune stromal cells [7]. The significant changes in multiple immune subpopulations reported here were accompanied by increases in markers of T cell activity, confirming that the net outcome of fulvestrant actions can counter some of the immunosuppressive effects of RT in the TME.

Activation of a type I IFN response has been shown to be necessary for RT-induced anti-tumor immunity in multiple preclinical cancer models, including ER negative breast cancer, and its cooperative interaction with ICIs [34–36]. As expected, in our study, RT potently induced this response in TC11 cells in vitro, as well as TC11 mammary tumors in vivo. However, whereas in vitro, fulvestrant did not alter the RT-induced rise in *Ifnβ* mRNA, it attenuated this response in vivo, even as it cooperated with RT to slow tumor regrowth and facilitated responses to the ICIs. The disparate behaviors of TC11 cells in vitro and TC11 tumors in vivo may result from effects of fulvestrant on immune or stroma tumor components in vivo or from indirect effects of these components on the response of TC11 cells to RT. For example, estrogen can upregulate IFNβ in myeloid cells [21, 44]. Moreover, estrogen has a complex relationship with NFκB, a regulator of *Ifnβ* transcription. Under inflammatory conditions, such that as observed in RT-treated tumors in vivo but not in standard culture conditions in vitro, estrogen can cooperate with this pathway [45].

The fulvestrant-induced delay in regrowth of irradiated ER+ mammary tumors was transient. By 25 days after RT, tumor growth had resumed, albeit at a slightly slower rate, and few differences in the cytokine milieu were observed among treatment groups. At this late timepoint, intratumoral concentrations of IFNγ in mice co-treated with RT+Fulv were not different from those in control animals, indicating that CD8+ T cell activity was once more suppressed. However, CCL2 and CXCL10 were significantly higher with the RT+Fulv combination at this time, demonstrating prolonged immunomodulation by the SERD following the perturbations initiated by RT. Elevation of these chemokines suggests that this co-treatment fuels ongoing recruitment of immune cells, including monocytes, CD8+ T cells and natural killer cells [41], and suggests mechanisms underlying immune escape.

In contrast to the lack of efficacy of the ICIs in combination with RT alone, RT in the context of fulvestrant permitted both anti-PD-L1 and anti-CTLA-4 to further slow tumor growth, confirming that the RT+Fulv treatment regimen remediated mechanisms that suppress the response to immune checkpoint blockade. Although the survival benefit that we observed in this experimental design was modest, our promising results with the combined therapies underscore the complementary actions of RT and anti-estrogens in the TME and the therapeutic potential of this strategy. In future studies, optimization of RT and fulvestrant dosing and timing, and additional examination of the mechanisms driving tumor escape, are warranted to improve outcomes and potentially to identify new immunologic targets [13].

Development of immunotherapies for patients with ER+ breast cancer is in its infancy. Ongoing exploration of the immune environments by single cell sequencing, mass cytometry, multiplex immunofluorescence and spatial resolution of communication networks is illuminating the mechanisms driving immunosuppression, differences among metastatic sites and responses to treatments of heterogeneous clinical ER+ breast cancers [7, 8]. These studies are revealing additional immune targets that may lead to efficacious treatments for these patients. For example, ER+ tumors with de novo resistance to aromatase inhibitors display elevated expression of the checkpoint proteins, LAG3 and IDO1 [46] that may be contributing to T cell exhaustion. Future studies can evaluate these other ICIs, and additional immune stimuli (e.g., intratumoral IL-2) to amplify clonal expansion and activation of effector T cells and potentially extend the benefit of combinatorial anti-estrogen and radiation therapy. We have shown benefit from these approaches in other tumor types [47–51]. Macrophages with immunosuppressive activities are particularly abundant in more aggressive ER+ tumors [38–40, 52], and patients with ER+ tumors where macrophages predominantly expressed an M1 phenotype had a better prognosis than those displaying more M2-like polarization [53, 54]. Robust interest across many cancers is fueling development of approaches to manipulate recruitment and/or modulate functional states of these cells [55, 56].

Anti-estrogens target multiple cell types within the ER+ TME, including not only tumor cells and multiple immune cells of both the lymphoid and myeloid lineages, but also many other stromal components, such as heterogeneous fibroblasts, adipocytes and endothelial cells, in a dynamic extracellular matrix, which can further modify the immune environment [7]. The current study demonstrates that the sum of anti-estrogen actions can favorably immunomodulate the ER+ breast cancer TME in the context of RT, an established adjuvant and palliative treatment, even when these tumors no longer depend on estrogen for growth and are resistant to anti-estrogen monotherapy. Combinatorial immunomodulatory approaches tailored to augment anti-tumor immunity for specific molecular subtypes of breast cancer hold the promise for enabling durable tumor regression for this devastating disease.

## Supplementary Information

**Additional file 1**. Antibodies and primers for qPCR utilized in these studies.

**Additional file 2**. Data supporting responsiveness of TC11 cells to 17β-estradiol and fulvestrant, flow cytometry gating strategies, and quantification of intratumoral Ly6C+/Ly6G+ cells.

## Data Availability

The datasets supporting the conclusions of this article are included within the article and its additional files.

## Abbreviations

PD1: Programmed death-1
ER+: Estrogen receptor positive
FFPE: Formalin-fixed paraffin-embedded
Fulv: Fulvestrant
ICI: Immune checkpoint inhibition
IFN: Interferon
MDSC: Myeloid derived suppressor cells
RT: Radiation therapy
SERD: Selective estrogen receptor downregulator
TME: Tumor microenvironment
TNBC: Triple negative breast cancer
Tregs: Regulatory T cells
