# Supplementary material for: Estrogen receptor blockade and radiation therapy cooperate to enhance the response of immunologically cold ER+ breast cancer to immunotherapy
Source: Breast Cancer Res. 2023 Jun 13;25:68. doi: 10.1186/s13058-023-01671-y (PMC10265911; doi:10.1186/s13058-023-01671-y)
Supplement: Supplementary file 1 — Additional file 1. Antibodies and primers for qPCR utilized in these studies. [file 13058_2023_1671_MOESM1_ESM.pdf]

Additional file 1 Table S1A. Antibodies used for chromogenic immunohistochemistry

Antigen	Vendor/ Catalog number	Clone	RRID	Dilution
CD8	ThermoFisher 14-0081-82	53-6.7	RRID:AB_467087	1:1000
ERα	Santa Cruz Biotechnology, Inc. sc-542	N/A	RRID:AB_631470	1:500
LY6C/LY6G	Abcam Ab25377	RB-6-8C5	RRID:AB_470492	1:200
PD-L1	Invitrogen 14-5982-82	MIH5	RRID:AB_467781	1:500
F4/80	Cell Signaling Technology 70076	D2S9R	RRID:AB_2799771	1:1000

Additional file 1 Table S1B. Antibodies used for multiplex immunofluorescence

Antigen	Vendor/ Catalog number	Clone	RRID	Dilution, incubation conditions	OPAL fluorophore
CDllB	Abcam Ab133357	EPR1344	RRID:AB_2650514	1:1500 15 min RT	540
F4/80	Abcam Ab6640	CI:A3-1	RRID:AB_1140040	1:50 45 min RT	520
CD86	Abcam ab119857	GL-1	RRID:AB_10902800	1:100 30 min RT	620
CD206	Abcam Ab64693	N/A	RRID:AB_1523910	1:1000 15 min RT	570
LY6C/LY6G	Invitrogen MAI-10401	RB-6-8C5	RRID:AB_11152791	1:100 ON 4°C	650

Additional file 1 Table S2. Antibodies used to stain cells from disaggregated tumors for flow cytometry.

Target	Fluorophore	Vendor	Clone
Live/Dead	GhostRed 780	Tonbo Biosciences	-
CD11b	Brilliant Violet 711	BioLegend	M1/70
CD3	FITC	Tonbo Biosciences	17A2
CD4	Brilliant Violet 785	BioLegend	RM4-5
CD4	APC	Tonbo Biosciences	RM4-5
CD45	Brilliant Violet 510	BioLegend	30-F11
CD45	PE-Cy7	Tonbo Biosciences	30-F11
CD8A	Alexa Fluor 700	BioLegend	53-6.7
CD8A	PE	Tonbo Biosciences	53-6.7
FOXP3	Brilliant Violet 421	BioLegend	MF-14
F4/80	PE-CF594	BD Biosciences	T45 2342
Ly6C	Brilliant Violet 605	BioLegend	HK1.4
Ly6G	Alexa Fluor 700	BioLegend	1A8

Additional file 1 Table S3A. Primer sequences used for qPCR (SYBR green)

Gene	Forward Primer	Reverse Primer
Cxcl10	CGCTGCAACTGCATCCATA	TAGGCTCGCAGGGATGATTTC
Icos	TCTTGTAAATACCCTGAGACTGTCC	TCGCAGAGGACTTCTCTCTCTC
Oas2	TAAGAGGCTGCTCCGATGGT	GACGTCAAGGTATGCATCTTGGT
Oas3	TTTCTCAGTCAAAGGCGTCCA	TCTATCCAGTGTTCTCCGTCTG

Additional file 1 Table S3B. TaqMan^{TM} predesigned gene expression assays

Gene	TaqMan Probe
Greb1	Mm00479269_m1
Hprt	Mm03024075_m1
Ifnβ	Mm00439552_s1
Ifnγ	Mm01168134_m1
Mhc1 (H2-K1)	Mm04208017_mH
Mx1	Mm00487796_m1
Nos2	Mm00440502_m1
Pdl1 (CD274)	Mm03048248_m1
