# Supplementary material for: Estrogen receptor blockade and radiation therapy cooperate to enhance the response of immunologically cold ER+ breast cancer to immunotherapy
Source: Breast Cancer Res. 2023 Jun 13;25:68. doi: 10.1186/s13058-023-01671-y (PMC10265911; doi:10.1186/s13058-023-01671-y)

Additional file Figure S1. Fulvestrant downregulates ERα expression and inhibits induction of estrogen target genes in ER+ TC11 tumor cells in vitro, and reduces uterine weight in vivo. (A) TC11 cells in culture were incubated with ethanol vehicle (control), 17β-estradiol (E2, 10nM) or fulvestrant (Fulv, 100nM) for 24h. (i) Cell lysates were fractionated by gel electrophoresis, and transferred to a PVDF membrane. The membrane was cut, and the upper portion probed with anti-ERα, and the lower portion probed with anti-ERK1/2 (loading control). Right, uncropped blot images. (ii) *Greb1* transcripts quantitated by qPCR (mean fold change ± SEM, n=3). (B) Fulvestrant (250 mg/kg sc weekly) or peanut oil vehicle (control) was administered to tumor bearing mice (see Fig. 1B), and efficacy was confirmed by assessment of uterine weight 32 days after initiation of treatment. (mean ± SEM, n=5; ****, p<0.0001).

A

i

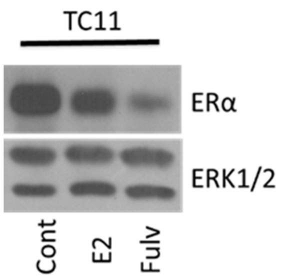

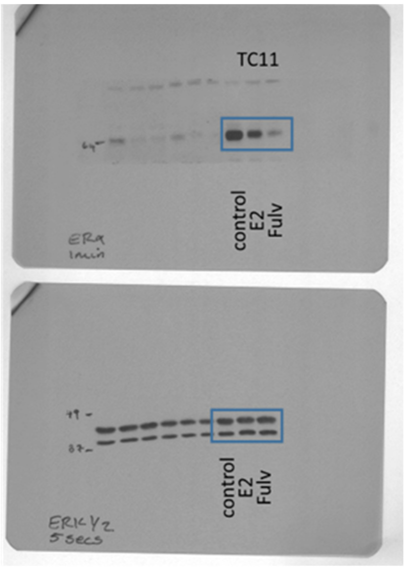

ii

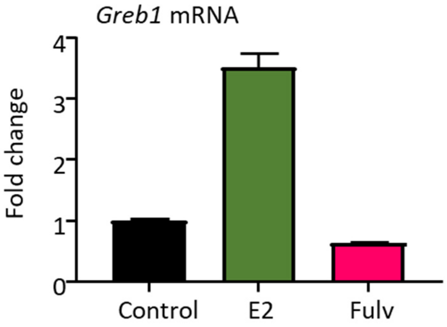

B

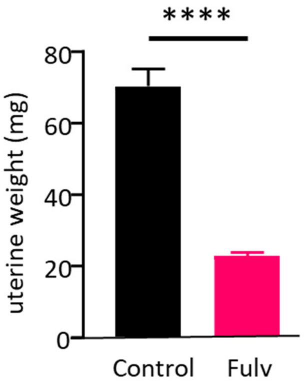

Additional file Figure S2.

A. Flow cytometry gating strategy used to interrogate innate immune subpopulations.

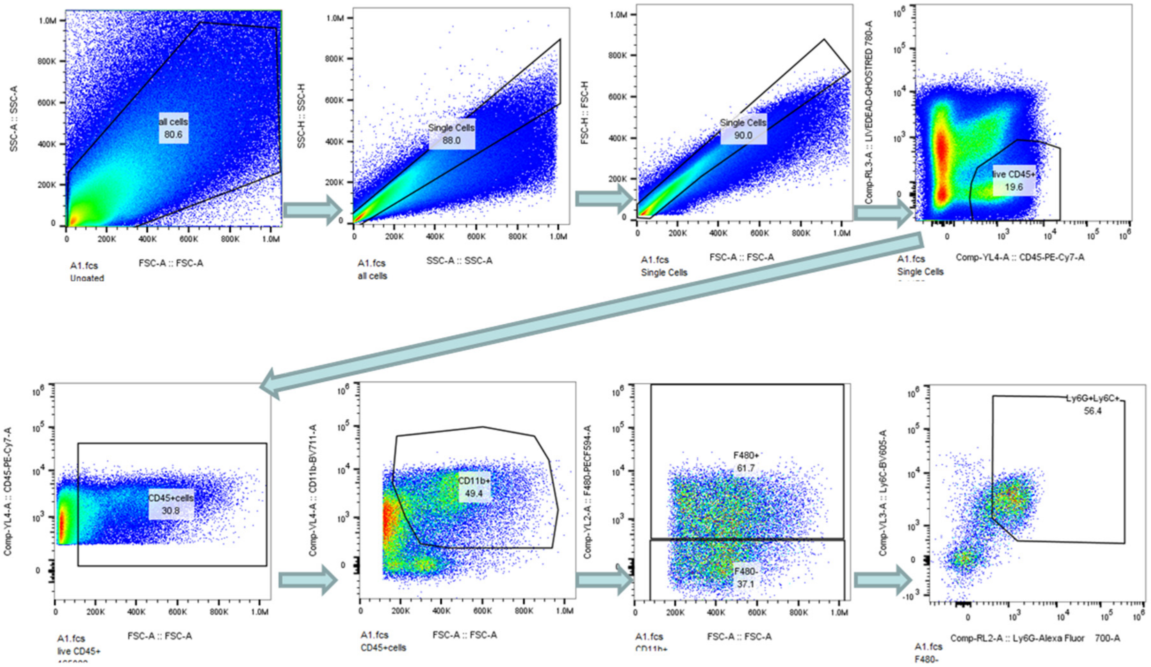

B. Flow cytometry gating strategy used to interrogate adaptive immune subpopulations.

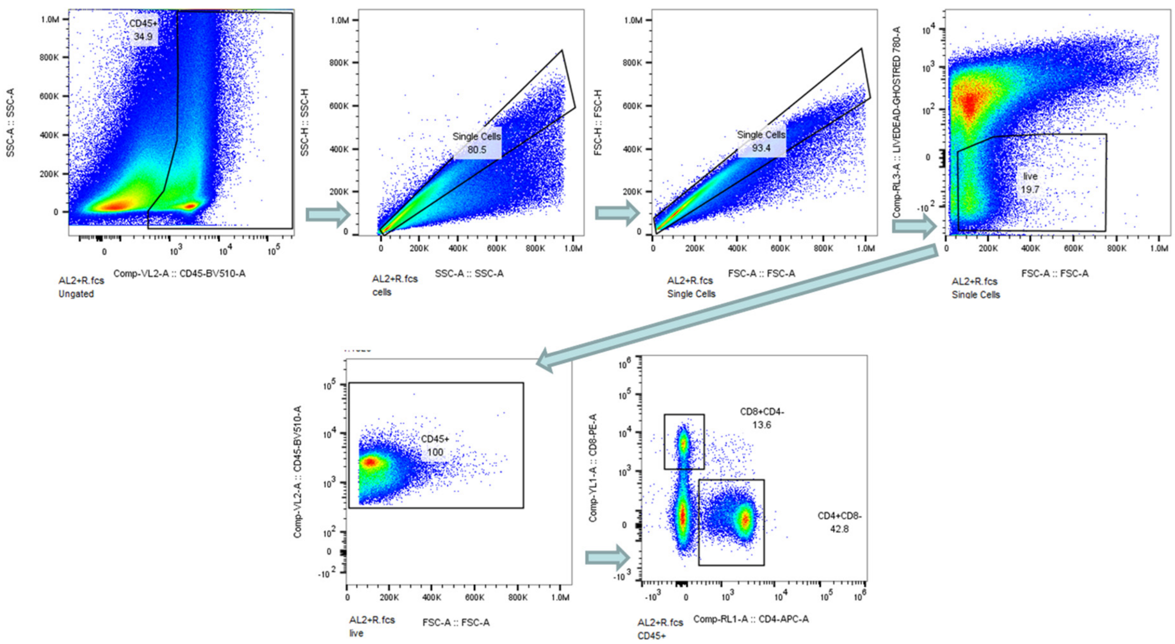

Additional file Figure S3. Fulvestrant reduces RT-recruited intratumoral Ly6C+/Ly6G+ cells. Animals were treated as shown in **Fig. 4A**, and effects of treatments examined 5 days after RT. Quantification of Ly6C+Ly6G+ cells by immunohistochemistry (mean ± SEM, n=5) (See **Fig. 4D**). Differences among treatments were determined by one way ANOVA, followed by Tukey post tests. *, P<0.05; **, P<0.01; ***, P<0.001; ****, P<0.0001.

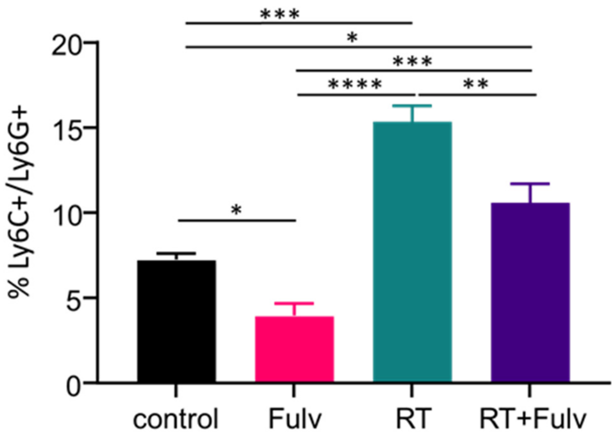

Additional file Figure S4. By day 25 after RT, tumor growth has resumed, and there are few long lasting changes in cytokines in the TME. Animals were treated as shown in **Fig. 6A.** Cytokine concentrations in tumor lysates collected from animals treated with vehicle, fulvestrant, RT, or RT + fulvestrant 53 days after transplantation (25 days after RT) were examined by multiplex immunoassays (see Materials and Methods). Individual tumors shown. With the exceptions of CCL2 (MCP1) and CXCL10 (IP-10) (**Fig. 6B**), no significant differences were observed between RT+ Fulv and other treatment groups at this time point.

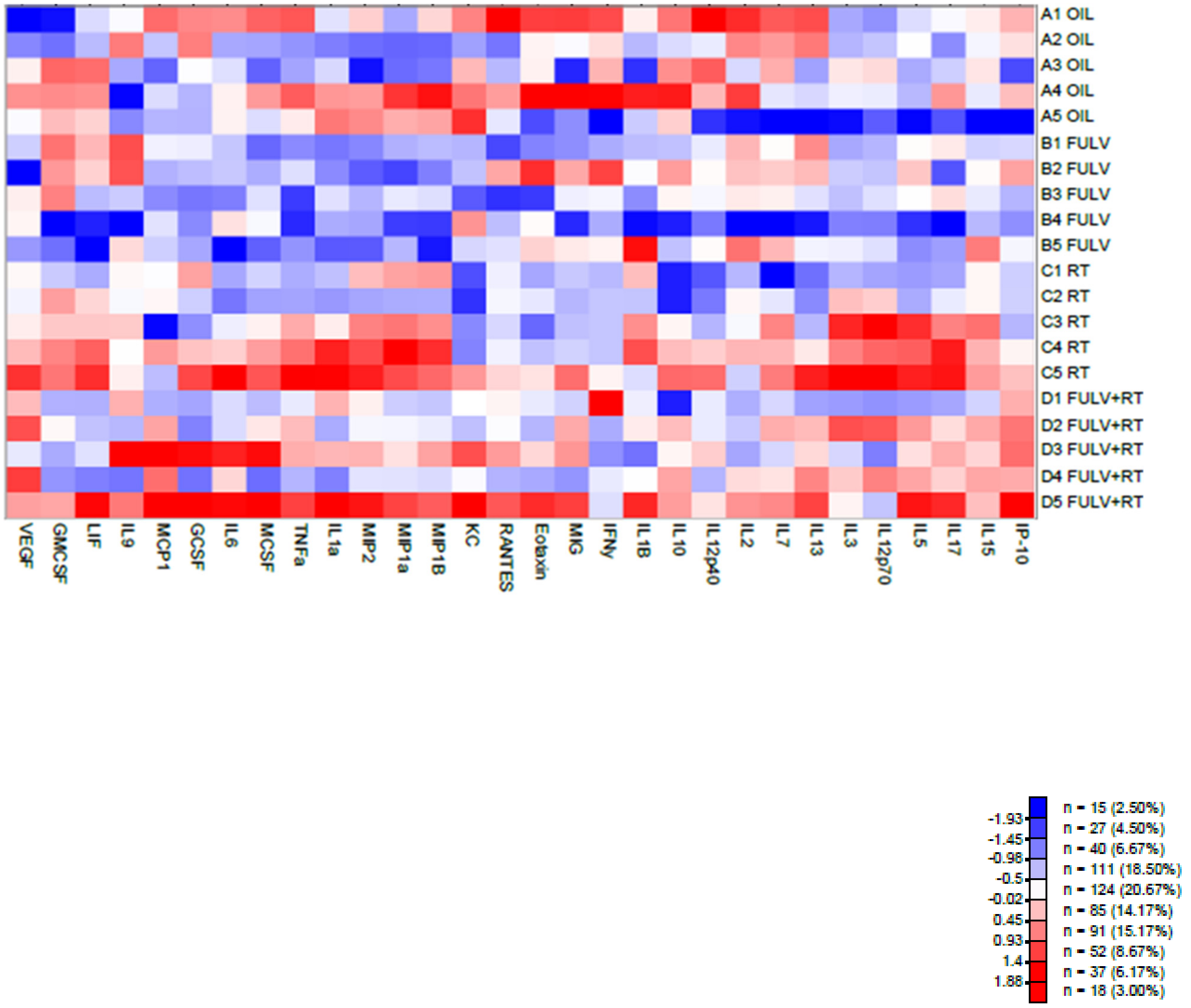

Supplement: Supplementary file 2 — Additional file 2. Data supporting responsiveness of TC11 cells to 17β-estradiol and fulvestrant, flow cytometry gating strategies, and quantification of intratumoral Ly6C+/Ly6G+ cells. [file 13058_2023_1671_MOESM2_ESM.pdf]
